# Cold-induced vasomotion changes in healthy individuals and patients after median or ulnar nerve repair

**DOI:** 10.1038/s41598-026-70936-1

**Published:** 2026-09-15

**Authors:** Hedvig Detert, Robin Mirdell, Erika Nyman, Erik Tesselaar, Simon Farnebo

**Affiliations:** 1 Department of Biomedical and Clinical Sciences, Faculty of Medicine and Health Sciences, Linköping University, Linköping, Sweden; 2 Department of Plastic Surgery, Hand Surgery, and Burns, Linköping University, Linköping, 58185 Sweden; 3 Department of Clinical Chemistry, Linköping University, Linköping, Sweden; 4 Department of Orthopedic and Hand Surgery, Faculty of Medicine and Health, Örebro University, Örebro, Sweden; 5 Department of Medical Radiation Physics, Department of Medical and Health Sciences, Linköping University, Linköping, Sweden; 6 Center for Medical Image Science and Visualization, Linköping University, Linköping, Sweden

**Keywords:** Vasomotion, Cold intolerance, Cold stress test, Microcirculation, Perfusion, Diseases, Medical research, Neurology, Neuroscience

## Abstract

This exploratory study investigated digital vasomotion during and after a standardized cold stress test in healthy subjects and patients previously treated for median or ulnar nerve injury. Twenty-five healthy individuals and 20 patients with a median or ulnar nerve injury were included. Finger perfusion was measured before and after a Cold Stress Test, using Laser Speckle Contrast Imaging. Vasomotion activity was quantified as the area under the curve of synchronized perfusion dips, and comparisons were made within and between the groups. Synchronized perfusion dips were observed in healthy participants both before and after cold provocation but were attenuated in patients with nerve injury. This suggests an impaired neural modulation of digital microvascular tone among nerve-injured patients. Among male patients with ulnar nerve injury, baseline perfusion was lower in the injured digit V than in the corresponding uninjured digit V (132 vs. 157 PU, *p* = 0.001), accompanied by reduced vasomotion activity (AUC 1.07 vs. 1.92, *p* = 0.010). In conclusion, neural mechanisms may be important in regulating vasomotion in the hand. Patients with a nerve trunk injury exhibit a different vasomotion pattern compared with healthy participants, supporting an important role of neural regulation in the alteration of digital vasomotion.

**Supplementary Information:**

The online version contains supplementary material available at https://doi.org/10.1038/s41598-026-70936-1.

## Introduction

Vasomotion has been described as the rhythmic oscillation in blood vessel tone, with spontaneous changes in the tone of blood vessels, occurring independently of heart rate and respiration^1^. This alters the lumen diameter and thereby modifies the blood flow, a process known as flow motion. Digital vasomotion refers to spontaneous, rhythmic changes in the tone of small blood vessels in the fingers, causing transient fluctuations in digital blood flow and perfusion. The phenomenon of vasomotion and flow motion has been investigated in vitro and in vivo using several techniques, such as laser Doppler flowmetry (LDF), and Laser Speckle Contrast Imaging (LSCI)^2^, in order to understand the physiological mechanisms behind the phenomenon. It has been stated that the oscillations in different frequencies originate from different parts of the cardiovascular system, such as the heart, the respiratory activity, and the endothelial and myogenic activity of the blood vessels.

Vasomotion is also altered in medical conditions such as diabetes^3^ and hypertension, and is largely influenced by the sympathetic nervous system. This suggests that neural mechanisms also impact the vasomotion, and thereby the perfusion of tissues. It is thus likely that nerve injuries can impair vasomotor regulation, leading to abnormal microcirculatory responses. This is particularly relevant in conditions such as Complex Regional Pain Syndrome (CRPS) and hand-arm vibration syndrome (HAVS), where nerve dysfunction contributes to a microvascular dysregulation that causes persistent symptoms in the form of vasoconstriction or abnormal vasodilation. Using dynamic contrast-enhanced magnetic resonance neurography, Mooshage et al. found associations between sciatic nerve perfusion parameters and small-fiber sensory function in patients with type 2 diabetes^4^. Similarly, Bäumer et al. demonstrated increased contrast uptake in peripheral nerves in patients with neuropathies compared with healthy controls, mainly reflecting increased blood–nerve barrier permeability rather than increased nerve blood volume^4^, further supporting an association between nerve pathology and altered intraneural microvascular function. However, despite the established influence of neural mechanisms on microvascular tone, the long-term effects of nerve injuries on digital vasomotion have remained poorly understood.

In patients with nerve injuries to the hand and upper limb, cold sensitivity is a common and bothersome symptom^5,6^. In cold sensitivity, the hand and fingers react to cold stimuli with pain, stiffness, sensory changes, and weakness, with or without color changes^7,8^. It has previously been proposed that microcirculatory changes might be a contributing mechanism for the development of cold sensitivity after peripheral nerve injury^8,9^. More severe cold sensitivity has been observed in patients with reduced sensory function after peripheral nerve repair^2,10^. The impact of nerve injuries on perfusion and vasomotion has been highlighted in studies showing differences in digital microvascular responses between median and ulnar nerve-innervated fingers during cold stress tests^11^.

Previous studies have investigated vasomotion using the LSCI to measure hand and nail bed perfusion, providing insight into normal and pathological responses to cold exposure^11–14^. It has been suggested that the physiological digital microvascular response to a standardized cold stress test(CST) may differ between fingers innervated by the median nerve (digits II and III) and by the ulnar nerve (digit V)^11^. Furthermore, exposure of one hand to cold has been shown to influence perfusion in the contralateral hand, suggesting that centrally mediated regulatory mechanisms are involved. Until now, the treatment options for cold sensitivity are sparse^15^, and understanding these differences is therefore crucial for improving clinical assessment and developing more effective treatment strategies for patients suffering from cold sensitivity following nerve injuries.

The aims of this exploratory physiologic study were to investigate whether there were any differences in vasomotion following a standardized CST between healthy test subjects and patients with nerve trunk injuries. We also aimed to investigate if there are differences in vasomotion between women and men. We hypothesized that patients with nerve trunk injuries would show reduced vasomotion compared to healthy controls, and that vasomotion would differ between sexes.

## Materials and methods

### Participants

**Fig. 1:**
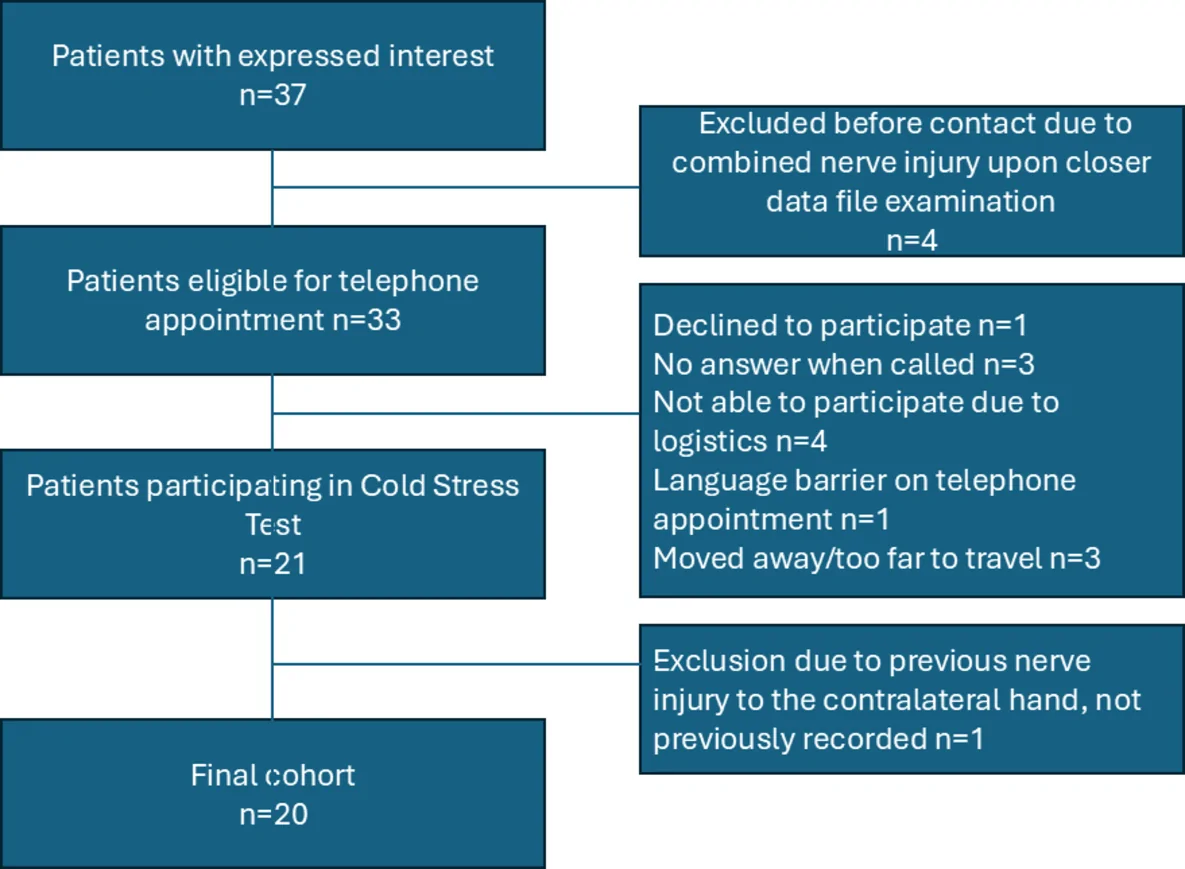
Showing the exclusion of patients participating in Cold Stress test.

Twenty patients with a previously treated median or ulnar nerve injury were included in this exploratory cross-sectional study. Recruitment was based on a questionnaire regarding cold sensitivity and nerve function, which also assessed interest in participating in a clinical study. Of 37 patients who expressed interest, 16 patients were excluded due to eligibility, logistics, or lack of response, and one was excluded because of a bilateral nerve injury (Fig. 1). Inclusion criteria were prior treatment for a median or ulnar nerve injury at Linköping University Hospital (2008–2019), proficiency in Swedish, and ability to attend the clinical trial. Both partial and complete injuries were accepted, whereof 50% (*n* = 10) had a complete injury and 50% had a partial injury. The level of injury was at the elbow or 10 cm proximal thereof in 25% of the cases (*n* = 5), in the forearm in 20% (*n* = 4), at wrist level in 30% (*n* = 6) and at hand level in 25% (*n* = 5). Recruitment began in 2020, but testing was postponed to 2022–2023 due to the COVID-19 pandemic.

Patient data were compared with 26 healthy participants from a previous study by our group^11^, conducted with the same CST protocol (except that the cold exposure was always on the right hand). One healthy male was excluded after post hoc visual inspection of the recordings because his vasomotion pattern showed markedly atypical inter-finger perfusion differences compared with the coordinated pattern observed in the other healthy participants (Fig. 3). No a priori quantitative threshold or statistical exclusion criterion had been defined; therefore, the exclusion was based on a qualitative assessment of the recording and not on a formal statistical rule. The reason for this atypical pattern could not be determined and may have reflected biological variability or technical factors, such as local vascular differences, region-of-interest placement, or motion. This post hoc exclusion is acknowledged as a limitation. The study was approved by the regional ethics committee (Regionala etikprövningsnämnden i Linköping) (Register number 2018/187 − 31), and all participants provided informed consent in accordance with the Declaration of Helsinki.

### Equipment

**Fig. 2:**
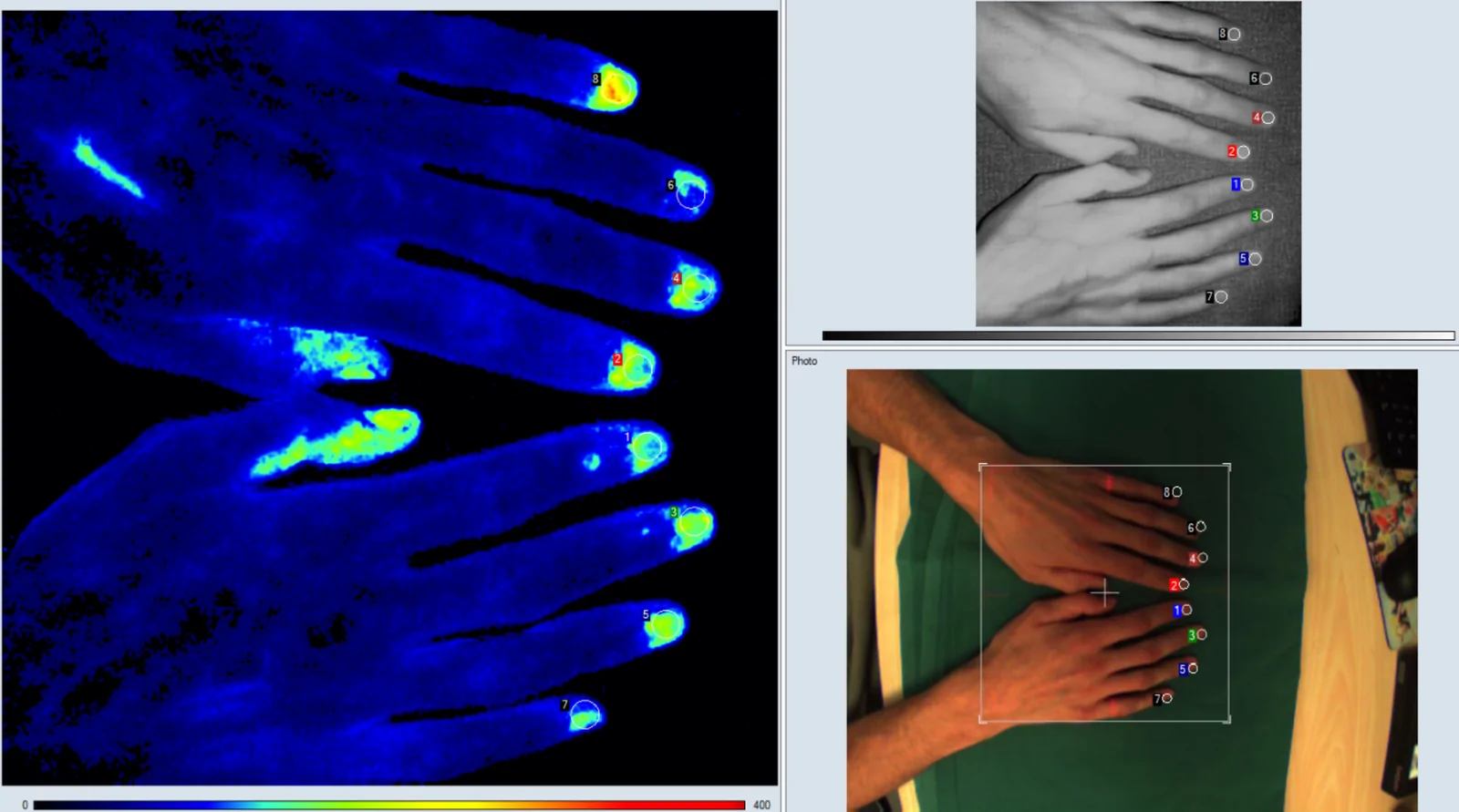
Describes the setup for the perfusion measurement. White circles + numbers show the regions of interest for each finger. The left half of the image (2a) shows a color-coded perfusion image with a perfusion scale at the bottom. In the upper right corner (2b), the intensity map of the infrared image is seen. In the lower right corner (2c), a normal image is shown.

To analyze the perfusion of the fingers on a microvascular level, LSCI (PeriCam psi System, Perimed AB Järfälla Sweden, https://www.perimed-instruments.com/products/pericam-imaging-systems/pericam-psi/), software PIMSoft version 1.5.4.8078was used. The LSCI uses a near-infrared laser, which operates at a wavelength of 785 nm and with a resolution of up to 33 μm/pixel. The images were acquired at a measurement distance of 30–35 cm with 5.3 images/second. At this distance, the resolution was around 0.8 mm/speckle pixel. The perfusion is reported in perfusion units (PU), an arbitrary value that reflects the concentration and mean velocity of red blood cells in the tissue rather than the flow. Higher perfusion values were visualized as red or yellow areas and lower perfusion correlates to blue and green areas in the LSCI software. (Fig. 2a). The LSCI system was calibrated according to the manufacturer’s guidelines.

### Cold stress test

A standardized Cold stress test (ISO 14835-1:2016), designed for the evaluation of peripheral vascular function in conditions following mechanical vibration and shock, such as Hand-Arm-Vibration-Syndrome (HAVS), was used^16^. The participants were asked to abstain from heavy physical exercise and caffeine intake for 24 h prior to the test. The participants were acclimatized to the room temperature for about 15 min while they were asked to complete a short questionnaire about their injury and experience of cold. During this time, measurements of sensation and power in the hands were performed. The study participant was then seated comfortably in an upright position with their arms and hands resting on a table in front of them. The participants’ feet were resting stably on the floor in order to avoid motion artefacts. The hands were placed close to each other with the dorsum up and with a small distance between the fingers (Fig. 2c). Under the hands, a green surgical sheet was placed, to reduce light reflections, and the LSCI camera was placed perpendicular to the hands at a distance of 30–35 cm. Thereafter, the setup was controlled, and the perfusion measurement was initiated. When 25 min had passed, the recording was paused, and the hand with a nerve injury was covered with a surgical latex glove to avoid evaporation effects. The injured hand was immersed in a water bath of 12 °C for 5 min while the registration of the perfusion was continued on the contralateral hand. After 5 min in the cold water, the recording was paused, and the hand was taken out. The surgical glove was removed, and the hand was returned to the table in the same position as it had been earlier. The registration process was then restarted and recorded for another 25 min. During the CST, the participants were asked about their experienced level of discomfort or pain at 1, 5, 6, 10, and 15 min after the cold exposure started, according to the NRS (Numerical Rating Scale). The regions of interest for each finger were manually adjusted after the recording, to match the same position on the nail bed before and after the cold stress test. With this methodology the LSCI has been shown to have excellent reproducibility to assess microvascular reactivity^17^.

### Vasomotion activity

**Fig. 3:**
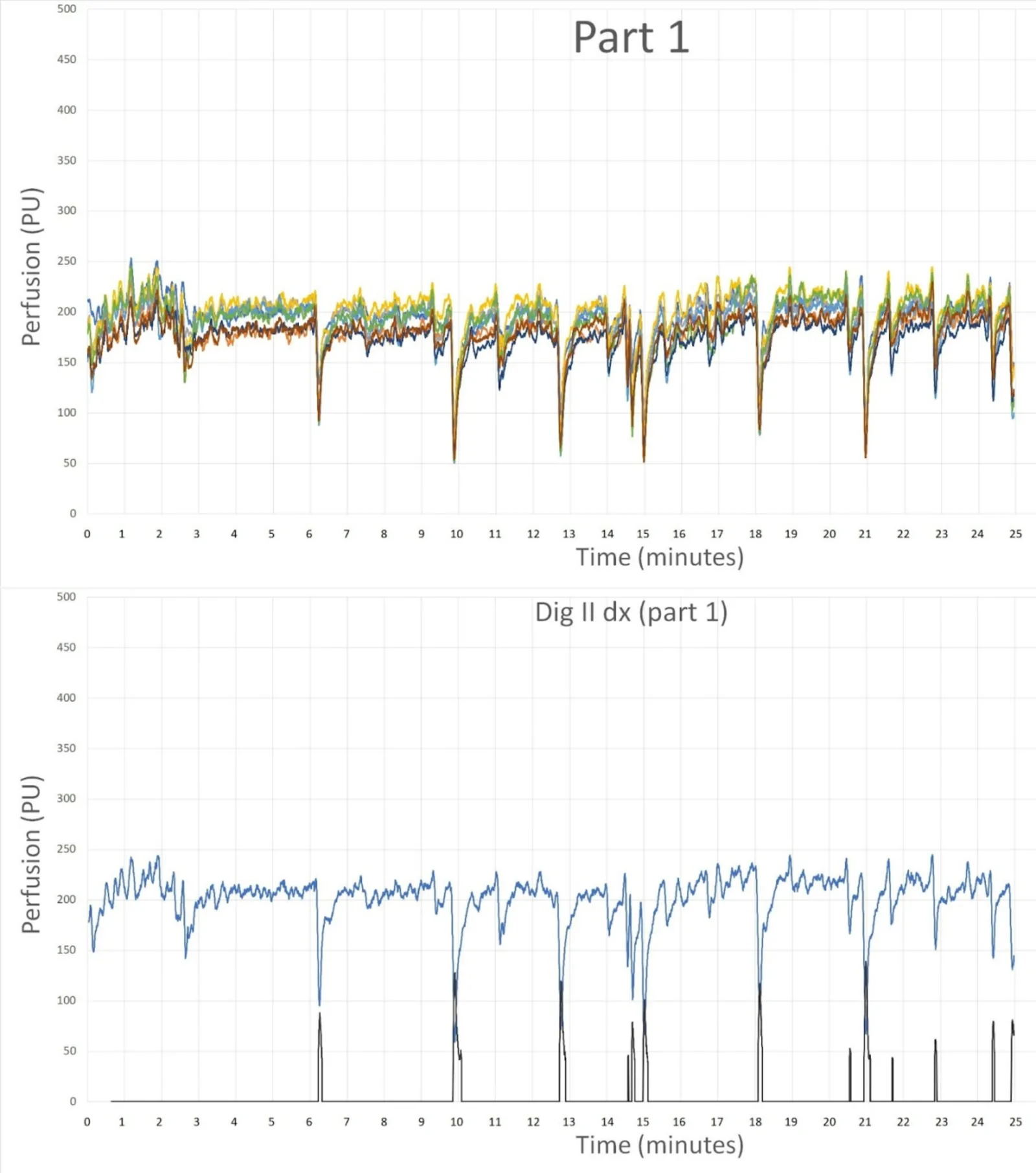
Baseline measurement from one of the healthy males (m_007). The graph shows the 4 s moving average of the perfusion signal from each finger: dark blue = dig II dx, orange = dig II sin, green = dig III dx, yellow = dig III sin, light blue = dig IV dx, and green = dig IV sin. The bottom graph shows the perfusion of dig II dx (dark blue) in combination with perfusion dip detection (black). The average AUC of this measurement was 3.74 PU, which is a typical value.

Vasomotion activity was measured using a previously described method that detects coordinated transient reductions in nail bed perfusion and expresses their magnitude as an area-under-the-curve (AUC) value. This corresponds to the average perfusion dip activity during the entire measurement. To achieve this, the baseline perfusion is first calculated, and when a sudden decrease is identified, a perfusion dip has occurred (Fig. 3). The area under the curve of an individual perfusion dip is calculated by comparing the measured perfusion value with an estimation of what the perfusion would have been had the perfusion dip not occurred. The estimation of this assumed perfusion is based on a percentile calculation of the previously recorded perfusion 36.4 s before the perfusion dip occurred. The method has previously been described in detail^2^.

### Data analysis

Data normality was tested using the Kolmogorov-Smirnov test. This showed that the vast majority of the data violated the assumption of normality, why non-parametric methods were used. Wilcoxon signed-rank tests were used for pairwise comparison of perfusion and vasomotion between digits II and V in healthy participants. Differences in perfusion and vasomotion between the sexes were analyzed with the Kruskal–Wallis test, and Dunn’s test with Bonferroni correction was used for between-group comparisons. Because of the low number of females with both median and ulnar nerve injury, further statistics were not done for these two groups. The number of males with median and ulnar nerve injury was considered sufficient for exploratory subgroup analysis. The Kruskal–Wallis test, and Dunn’s test with Bonferroni correction was used for comparisons with the healthy males. Correlation between baseline perfusion (affected digit when applicable), baseline AUC, and perfusion after cold-water provocation and pain (NRS) was done using Spearman’s rank correlation. The association between Body Mass Index (BMI), age and PU/AUC in the healthy controls was also investigated using Spearman’s rank correlation. Bonferroni correction was applied to the p-values of the Spearman’s rank correlation. The BMI difference between healthy males and females was tested with Student’s t-test. Differences in sensory function with the Semmes-Weinstein monofilament test were tested with the Mann-Whitney U-test. P-values ≤ 0.05 were regarded as significant.

## Results

The study population consisted of 25 healthy participants and 20 patients with a median or ulnar nerve injury. The healthy participants were 13 women and 12 men with a median age of 30 [IQR 11] years and BMI 24 (IQR 5). The median age of patients with nerve injuries was 52 [IQR 24] years, with a median of 9 [IQR 8] years from injury and median BMI of 26 [IQR 5]. The nerve injury group consisted of 4 women and 16 men, of whom 9 patients had median nerve injuries and 11 patients had ulnar nerve injuries. Of the nerve injury patients, 50% had a complete injury (*n* = 10), the remaining (*n* = 10) had a varying degree of partial nerve injuries. In the nerve injury group, 60% (*n* = 12) had injured their non-dominant hand and 5% (*n* = 1) were left-handed. The sensory function after injury was measured using Semmes-Weinstein monofilament test, which was a median of 4.31 in the injured finger related to the injured nerve (dig 5 for ulnar nerve injuries and dig 2 for median nerve injuries), and 2.83 in the non-injured finger (*p* = 0.002), which indicates a reduced protective sensation in the injured finger. The motor function after nerve injury was measured using a Pinch Grip Gouge and a Jamar dynamometer. The median pinch grip was 8.0 kg in the injured hand and 11.0 kg in the non-injured hand in the ulnar nerve injuries, and 7.0 kg in the injured hand and 9.8 kg in the non-injured hand in the median nerve injuries. In the ulnar nerve group, the median Jamar strength grip was 26.0 kg on the injured side and 50.0 kg on the non-injured side. In the median nerve group, the median Jamar grip strength was 44.0 kg in the injured hand and 46.5 kg in the non-injured hand. The median pinch grip in the entire group was 7.8 kg for the injured hand and 10.5 kg in the non-injured hand. The median Jamar strength was 42.0 kg for the injured hand and 49.5 kg in the non-injured hand for the entire group. The women had the highest reported pain at five minutes of cold provocation, NRS 4. The men in the nerve injury group had the median highest reported pain at five minutes of cold provocation of NRS 5.5. All participants tolerated the CST. In ulnar nerve injuries there was a negative correlation between NRS and the perfusion in digit V of the injured hand in part 1 and 3 of the measurement, r -0.67 (*p* < 0.001) and r -0.73 (*p* < 0.001), respectively.

In healthy participants, baseline perfusion was lower in women than in men, 140 PU vs. 159 PU (*p* = 0.050, Table 2). The baseline perfusion values showed a wider IQR in women than in men, 81–206 PU vs. 135–176 PU.

**Table 1:** Median perfusion values (PU) and vasomotion (AUC) for 13 healthy females and 12 healthy males with interquartile range within brackets. * indicates a significant difference between dig II and dig V at the same point in time. Part 1 consists of baseline measurement, part 2 the cold-water provocation, why there is no measurement data for the provoked hand, and part 3 is the recovery phase.

Healthy females	Dig II (PU)	Dig V (PU)	Dig II (AUC)	Dig V (AUC)
Part 1 (both hands)	149 (89–207) *p < 0.001	130 (77–202)	1.83 (1.30–4.78) *p < 0.001	3.58 (2.68–5.54)
Part 2 (provoked)	n/a	n/a	n/a	n/a
Part 2 (unprovoked)	65 (46–90)	54 (43–88)	1.64 (0.13–3.13) *p = 0.050	2.12 (0.72–4.80)
Part 3 (provoked)	95 (59–162)	77 (54–161)	1.19 (0.24–2.92)	2.32 (0.52–3.44)
Part 3 (unprovoked)	119 (56–176)	115 (51–163)	3.09 (1.07–4.58) *p = 0.005	4.07 (2.52–7.84)
Healthy males				
Part 1 (both hands)	166 (138–183) *p < 0.001	151 (134–171)	5.37 (3.20–7.70) *p = 0.021	6.32 (5.05–9.15)
Part 2 (provoked)	n/a	n/a	n/a	n/a
Part 2 (unprovoked)	91 (80–128)	98 (84–124)	5.32 (3.52–8.08)	6.27 (3.28–12.2)
Part 3 (provoked)	92 (68–139)	101 (75–133)	2.87 (2.43–5.28)	3.95 (2.58–6.59)
Part 3 (unprovoked)	142 (123–165) *p = 0.007	129 (119–152)	8.29 (5.47–9.90) *p = 0.041	8.70 (6.44–14.0)

**Table 2:** Median perfusion values (PU) and vasomotion (AUC) for 13 healthy females and 12 healthy males with interquartile range within brackets. * indicates a significant difference between the sexes at the same point in time. Part 1 consists of baseline measurement, part 2 the cold-water provocation why there is no measurement data for the provoked hand, and part 3 is the recovery phase.

Healthy test persons	PU - female	PU - male	AUC - female	AUC – male
Part 1 (both hands)	140 (81–206) *p = 0.050	159 (135–176)	3.00 (1.62–5.17) *p = 0.002	5.93 (3.43–8.11)
Part 2 (provoked)	n/a	n/a	n/a	n/a
Part 2 (unprovoked)	62 (46–88)	96 (82–125)	1.95 (0.38–4.64)	6.05 (3.52–10.1)
Part 3 (provoked)	88 (56–161)	100 (72–136)	1.46 (0.37–3.22)	3.05 (2.58–5.95)
Part 3 (unprovoked)	119 (55–167)	137 (119–156)	3.45 (2.03–5.80) *p = 0.001	8.44 (5.89–11.5)

Significant vasomotion differences between women and men in the normal healthy population, AUC 3.0 in women vs. 5.93 in men (*p* = 0.003) before performing a CST were found (Table 2). During recovery, AUC in the unprovoked hand was also significantly higher in men than in women, 8.44 vs. 3.45 (*p* = 0.001, Table 2).

Furthermore, a significant difference between fingers innervated by the median nerve compared to fingers innervated by the ulnar nerve in both women and men before performing a CST was found (Table 1) (*p* < 0.001 in women, *p* = 0.021 in men). At baseline, median perfusion values for digit II and digit V were 149 PU and 130 PU in healthy women, and 166 PU and 151 PU in healthy men, respectively (Table 1). Intermittent synchronized perfusion decreases were observed during baseline recordings, as illustrated in Fig. 3.

During cold provocation, perfusion in the unprovoked hand was lower in women than in men, 62 PU vs. 96 PU (Table 2). The IQR was wider in women than in men, 46–88 PU vs. 82–125 PU. During recovery, perfusion in the unprovoked hand was 119 PU in women and 137 PU in men (Table 2).

There was also a significant difference between the ulnar nerve innervated digit 5 in the injured hand compared to the uninjured hand in male patients with ulnar nerve injury (Table 3), both seen as perfusion units PU 132 (injured) vs. 157(uninjured) (*p* = 0.001) and vasomotion AUC 1.07 (injured) vs. 1.92 (uninjured) (*p* = 0.010).

**Table 3:** Median perfusion (PU) and vasomotion (AUC), and range (interquartile range for males) within brackets in two female and nine male patients with ulnar nerve injury. Median PU and AUC are also shown for 13 healthy females and 12 healthy males with interquartile range within brackets. Part 1 consists of baseline measurement, part 2 the cold provocation why there is no measurement data for the provoked hand, and part 3 is the recovery phase. * indicates a significant difference compared to healthy controls.

Females with ulnar nerve injury					
	Dig II	Dig II	Healthy controls	Dig V	Dig V (uninjured)
PU	(injured)	(uninjured)		(injured)	
Part 1	142 (99–175)	136 (121–212)	140 (81–206)	168 (108–187)	166 (122–183)
Part 2	n/a	109 (103–115)	62 (46–88)	n/a	91 (63–118)
Part 3	83 (39–129)	111 (95–173)	Provoked:	103 (29–173)	125 (59–151)
			88 (56–161)		
			Unprovoked:		
			119 (55–167)		
AUC					
Part 1	0.74 (0.29–1.20)	1.96 (1.49–2.46)	3.00 (1.62–5.17)	0.56 (0.16–0.96)	2.14 (1.91–2.38)
Part 2	n/a	1.84 (0.48–3.19)	1.95 (0.38–4.64)	n/a	3.30 (2.37–4.24)
Part 3	1.16 (0.81–1.52)	0.91 (0.78–1.04)	Provoked:	1.97 (0.59–3.35)	4.04 (3.12–4.97)
			1.46 (0.37–3.22)		
			Unprovoked:		
			3.45 (2.03–5.80)		
Males with ulnar nerve injury					
	Dig II	Dig II	Healthy controls	Dig V	Dig V (uninjured)
PU	(injured)	(uninjured)		(injured)	
Part 1	139 (119–166)	143 (126–161)	159 (135–176)	132 (75–152)	157 (126–184)
				*p = 0.001	
Part 2	n/a	115 (77–150)	96 (82–125)	n/a	126 (75–170)
Part 3	131 (59–147)	135 (109–147)	Provoked:	85 (63–135)	148 (101–169)
			100 (72–136)		
			Unprovoked:		
			137 (119–156)		
AUC					
Part 1	0.19 (0.06–1.67)	0.40 (0.09–1.59)	5.93 (3.43–8.11)	1.07 (0.15–2.22)	1.92 (0.28–3.60)
				*p = 0.010	
Part 2	n/a	0.34 (0.00–1.95)	6.05 (3.52–10.1)	n/a	1.75 (0.05–3.87)
Part 3	0.30 (0.08–2.38)	0.31 (0.00–3.32)	Provoked:	1.07 (0.12–1.57)	2.40 (0.34–5.11)
			3.05 (2.58–5.95)		
			Unprovoked:		
			8.44 (5.89–11.5)		

In male patients with median nerve injury, no significant difference in baseline perfusion was found between the injured digit II and healthy male controls, 152 PU vs. 159 PU (Table 4). However, AUC values were significantly lower in digit II of the uninjured hand at baseline compared with healthy male controls, 0.59 vs. 5.93 (*p* = 0.029, Table 4), and during recovery, 1.31 vs. 8.44 (*p* = 0.022, Table 4). AUC in digit V of the uninjured hand was also lower at baseline compared with healthy male controls, 0.80 vs. 5.93 (*p* = 0.033, Table 4).

**Table 4:** Median perfusion (PU) and vasomotion (AUC), and range (interquartile range for males) within brackets in two female and seven male patients with median nerve injury. Median PU and AUC is also shown for 13 healthy females and 12 healthy males with interquartile range within brackets. Part 1 consists of baseline measurement, part 2 the cold-water provocation why there is no measurement data for the provoked hand, and part 3 is the recovery phase. * indicates a significant difference compared to healthy controls.

Females with median nerve injury					
PU	Dig II (injured)	Dig II (uninjured)	Healthy controls	Dig V (injured)	Dig V (uninjured)
Part 1	125 (66–144)	136 (74–170)	140 (81–206)	135 (50–185)	142 (34–203)
Part 2	n/a	97 (60–134)	62 (46–88)	n/a	82 (31–133)
Part 3	59 (33–94)	80 (39–164)	Provoked: 88 (56–161) Unprovoked: 119 (55–167)	52 (22–85)	64 (21–154)
AUC					
Part 1	0.65 (0.46–0.84)	4.92 (3.95–5.88)	3.00 (1.62–5.17)	2.41 (1.36–3.45)	2.56 (2.28–2.85)
Part 2	n/a	0.64 (0.01–1.27)	1.95 (0.38–4.64)	n/a	2.60 (0.03–5.16)
Part 3	0.69 (0.00–1.38)	1.80 (0.08–3.52)	Provoked: 1.46 (0.37–3.22) Unprovoked: 3.45 (2.03–5.80)	1.04 (0.00–2.08)	3.22 (0.12–6.33)
Males with median nerve injury					
PU	Dig II (injured)	Dig II (uninjured)	Healthy controls	Dig V (injured)	Dig V (uninjured)
Part 1	152 (112–172)	151 (128–164)	159 (135–176)	152 (128–160)	143 (110–165)
Part 2	n/a	110 (89–140)	96 (82–125)	n/a	101 (79–129)
Part 3	96 (69–142)	150 (108–167)	Provoked: 100 (72–136) Unprovoked: 137 (119–156)	115 (87–142)	136 (89–152)
AUC					
Part 1	0.41 (0.00–2.03)	0.59 (0.04–1.23) *p = 0.029	5.93 (3.43–8.11)	1.21 (0.23–4.34)	0.80 (0.33–1.86) *p = 0.033
Part 2	n/a	0.08 (0.00–2.72)	6.05 (3.52–10.1)	n/a	1.78 (0.18–4.78)
Part 3	1.73 (0.21–2.77)	1.31 (0.14–2.38) *p = 0.022	Provoked: 3.05 (2.58–5.95) Unprovoked: 8.44 (5.89–11.5)	3.19 (0.68–3.55)	3.10 (1.59–4.91)

Data availability statement:

The original data presented in the study are included in the article. The datasets used and analyzed during the current study are available from the corresponding author on reasonable request.

Analysis of the healthy population also revealed an association between BMI and perfusion, but no correlation was found for BMI and AUC values. In healthy men, the correlation was negative, r -0.59 (*p* < 0.001), while a positive correlation was seen in women, r 0.43 (*p* = 0.041). BMI was found to be normally distributed in both groups and there was no significant difference between the included women, 23.0 ± 3.3 (mean ± SD), and the men, 25.7 ± 4.3 (*p* = 0.101). Perfusion was not correlated to age r -0.11 (*p* = 0.300), but AUC-values showed a negative correlation with age, r -0.35 (*p* < 0.001).

## Discussion

Our exploratory study showed that among nerve injury groups, individuals with ulnar nerve injury demonstrated significantly reduced baseline perfusion and AUC values in the injured digit compared to healthy subjects. These hypothesis-generating data indicates that individuals with median nerve injury will display a slightly different vasomotion pattern compared to those with an ulnar nerve injury. Baseline perfusion of the injured digit was not significantly different from that of healthy controls. However, the baseline AUC was reduced in digits II and V of the non-injured hand. As in individuals with ulnar nerve injury, individuals with median nerve injury had a reduced AUC value in digit II of the uninjured hand throughout the entire measurement (Table 4). When comparing healthy and injured groups, injured digits consistently exhibited significantly lower AUC values at baseline and showed a delayed recovery following cold exposure. Ulnar nerve injuries were associated with greater reductions in perfusion compared to median nerve injuries, indicating a more profound impact on vasomotor regulation.

We could also show differences in perfusion between healthy women and men. Women had a larger variation in baseline perfusion, suggesting it was not uncommon for the women to be in a vasoconstrictive state during the baseline measurement^18^. However, some women had a much higher baseline perfusion than the average man, which might be related to hormonal factors such as estrogen^19,20^. This is in line with previous studies that have shown that women show different patterns for vascular reactivity to a cold challenge, depending on their hormonal status^19,20^, considering that our population includes both pre- and postmenopausal women, with and without oral contraceptives. In comparison, men had a relatively homogenous baseline perfusion but showed much higher AUC values than women; both during the baseline measurement and in the unprovoked hand after cold provocation. Women also had slightly lower perfusion and much higher variation than men in the unprovoked hand, suggesting that women are more likely to respond with extensive contralateral vasoconstriction.

Despite these seemingly large differences between women and men, there is also a general pattern of perfusion response during a CST. During baseline measurement, all fingers have similar perfusion with occasional synchronized decreases in perfusion. Perfusion dips seem to be more common in men compared to in women. Since perfusion dips constitute a sudden decrease in perfusion, they cannot occur if the microcirculation is in a primarily vasoconstrictive state, explaining why perfusion dips are sometimes absent in healthy women^18^. After cold provocation, the perfusion will decrease primarily in the provoked hand, but also to a lesser degree in the contralateral hand^11,21^. For men, the AUC difference between the provoked and unprovoked hand was also significantly different, whereas it was not for women. These differences, especially the vasoconstrictive tendencies of some women, might explain the increased prevalence of cold sensitivity in women.

We also found an association between BMI and baseline perfusion in healthy controls. Women showed a correlation between perfusion and BMI, whereas perfusion was inversely correlated to BMI in men. Previous findings by Giurgea et al. also showed a similar correlation between BMI and perfusion as measured with laser Doppler imaging in mainly female patients with Raynaud’s phenomenon (approximately 80% women)^22^. However, because of the small sample size in our study, the exact implications of these differences between women and men should be interpreted with caution.

As in previous findings, we identified synchronized perfusion dips in most healthy individuals during baseline measurements. These dips, which appear as transient reductions in perfusion, are irregular but synchronized across digits. The presence of these dips suggests an underlying neural regulatory mechanism, which is further supported by our previous observation that a digital nerve block can completely abolish these dips. As might be expected, individuals with nerve injuries exhibited less pronounced perfusion dips than healthy controls, consistent with altered vasomotor regulation, although age and comorbidity may also have contributed.

In the ulnar nerve injury group, a significant negative correlation was observed between perfusion and pain after cold exposure. Participants with higher perfusion had a lower NRS score, indicating that ulnar nerve injuries that regained better microvascular function over time exhibited less pain when exposed to a cold stimulus and vice versa. This correlation was, however, not seen in median nerve injuries. It has previously been shown that patients with median nerve trunk injuries generally experience less cold-related pain than those with ulnar nerve injuries^23,24^. This effect is likely due to several factors, including differences in sensory fiber composition and autonomic function between these two nerves. Our findings highlight the role of neural mechanisms in vasomotion and perfusion. Previous studies have shown that cold-induced perfusion dips in the microcirculation can be abolished by a digital nerve block, confirming that neural control plays a crucial role in regulating vasomotion. The impact of nerve injuries on vasomotion may therefore be partially explained by disruptions in autonomic nerve function. Additionally, our study aligns with findings suggesting that patients with ulnar nerve injuries may experience greater cold intolerance than those with median nerve injuries. This may be attributed to differences in autonomic innervation and vascular responsiveness between these nerves.

Although our study may contribute to the general knowledge of the interplay between nerve function and distal microcirculation, several limitations should be noted. Although all patients who were surgically treated for a nerve trunk injury at our department were invited to participate in the project, our final sample size was relatively small. This was because the present study was a single center study, only including actively volunteering participants. One can speculate that patients with more severe cold sensitivity are more prone to avoid participation, as they wish to avoid the discomfort from undergoing CST. It is possible that such an inclusion bias might skew the material to participants with lower degrees of cold sensitivity. It is also a possibility that some patients choose to participate and might exaggerate their symptoms in order to try to get more follow-up or extra help with their nerve injuries.

Also, our material consists of patients with both complete and partial nerve injuries, introducing a heterogeneity in the material that may affect the severity of the effects of the nerve injuries, especially as a partial injury may, or may not involve fascicles that include autonomic fibers. A partial injury may thus spare these fascicles, leading to preserved vasomotor function, while a complete injury will not. Also, anatomical variations, such as cross-innervation between nerve trunks (i.e. Martin-Gruber connections) may provide autonomic contribution to injured dermatomes, especially in partial injuries. The mechanism of injury and the time from injury also differ between the nerve injury participants.

The injured group also consists of participants of a higher age and more comorbidities than the healthy subjects. The age difference between patients (median 52 years) and healthy controls (median 30 years) is not neglectable when considering how it affects vasomotion, regardless of nerve injury, where a higher age is likely to influence the vasomotion through both structural and functional changes. Ageing is believed to increase vascular stiffness^25^ through elastin fragmentation and collagen accumulation and impairs endothelial function through oxidative stress and reduced nitric oxide bioavailability^26^. Ageing is also seen to alter sympathetic regulation of cutaneous blood flow^27^ as well as through altered pathway-dependent signaling^28,29^ In our material, perfusion was not correlated with age, but AUC-values showed a negative correlation with age. However, this does not exclude confounding, and the effects of nerve injury, age and comorbidity cannot be separated in this small cohort. The patient–control differences should therefore be interpreted as exploratory rather than attributable to nerve injury alone. Future studies should use prospectively recruited age-matched controls. One could also consider matching the tested hand between the patients and the control group, even though this may have a smaller influence on the results. The testing of healthy controls and patients could also have been closer in time but were delayed due to the covid-19 pandemic.

The subgrouping of the different nerve injuries also constitutes a limiting factor, considering the resulting groups are small. A deeper analysis between men and women in the respective nerve groups was not possible to perform, considering the small number of women. However, the analysis in the nerve subgroups was considered valuable as they confirmed earlier studies that indicate more severe cold-related problems after ulnar nerve injury than after median nerve injury. One additional shortcoming with the study design is that the healthy controls came from a previous study, and the protocol was not fully identical because cold exposure in the controls was always on the right hand, whereas in patients, it was the injured hand.

Furthermore, the potential influence of psychological factors on subjective reports of cold sensitivity cannot be ruled out. Future studies should aim to include larger, more homogenous patient groups and investigate potential therapeutic interventions to mitigate cold sensitivity following nerve repair. Such a study would have to use a multi-center approach as the number of patients with complete nerve trunk injuries is relatively small in our society, and this is a patient group that may be difficult to include in long-term follow-ups.

## Conclusion

In conclusion, the findings from this exploratory study are consistent with altered vasomotor regulation. However, the patient cohort was small, and the results should therefore be regarded as hypothesis-generating, especially since the patient group was older and had more comorbidities than the healthy controls. Although the independent contribution of nerve injury cannot be established, the observed differences in microvascular responses between healthy individuals and patients with some nerve injuries highlight the importance of considering potential altered neural contributions to vasomotor regulation in clinical assessments. Further research is needed to develop targeted treatments aimed at improving microcirculatory function and alleviating cold sensitivity in patients with nerve injuries.

## Supplementary Information

Below is the link to the electronic supplementary material.

Supplementary Material 1

## Data Availability

The original data presented in the study are included in the article. The datasets used and analyzed during the current study available from the corresponding author on reasonable request.

## Abbreviations

CST: Cold Stress Test
NRS: Numerical Rating Scale
LSCI: Laser Speckle Contrast Imaging
LDF: Laser Doppler Flowmetry
CIVD: Cold Induced Vasodilatation
ROI: Regions of Interest
Dig: Digit
HAVS: Hand-arm-vibration syndrome
CL: Contralateral
PU: Perfusion units
